# Omicron variant susceptibility to neutralizing antibodies induced in children by natural SARS-CoV-2 infection or COVID-19 vaccine

**DOI:** 10.1080/22221751.2022.2035195

**Published:** 2022-02-10

**Authors:** Lin-Lei Chen, Gilbert T. Chua, Lu Lu, Brian Pui-Chun Chan, Joshua Sung-Chih Wong, Calvin Chit-Kwong Chow, Tak-Ching Yu, Agnes Sze-Yin Leung, Shu-Yan Lam, Tak-Wai Wong, Hing-Wai Tsang, Ian Chi-Kei Wong, Kwok-Hung Chan, Kwok-Yung Yuen, Patrick Ip, Mike Yat-Wah Kwan, Kelvin Kai-Wang To

**Affiliations:** a State Key Laboratory for Emerging Infectious Diseases, Department of Microbiology, Li Ka Shing Faculty of Medicine, Carol Yu Centre for Infection, The University of Hong Kong, Hong Kong, People’s Republic of China; b Department of Pediatrics and Adolescent Medicine, Li Ka Shing Faculty of Medicine, The University of Hong Kong, Hong Kong, People’s Republic of China; c Department of Pediatrics and Adolescent Medicine, The Hong Kong Children’s Hospital, Hong Kong, People’s Republic of China; d Department of Pediatrics and Adolescent Medicine, Princess Margaret Hospital, Hong Kong, People’s Republic of China; e Department of Pediatrics and Adolescent Medicine, United Christian Hospital, Hong Kong, People’s Republic of China; f Department of Pediatrics and Adolescent Medicine, Pamela Youde Nethersole Eastern Hospital, Hong Kong, People’s Republic of China; g Department of Pediatrics, Prince of Wales Hospital, Chinese University of Hong Kong, Hong Kong, People’s Republic of China; h Department of Pediatrics and Adolescent Medicine, Tuen Mun Hospital, Hong Kong, People’s Republic of China; i Department of Pediatrics and Adolescent Medicine, Alice Ho Miu Ling Nethersole Hospital, Hong Kong, People’s Republic of China; j Department of Pharmacology and Pharmacy, Li Ka Shing Faculty of Medicine, Centre for Safe Medication Practice and Research, The University of Hong Kong, Hong Kong, People’s Republic of China; k Laboratory of Data Discovery for Health (D24H), Hong Kong Science and Technology Park, Hong Kong, People’s Republic of China; l School of Pharmacy, Research Department of Practice and Policy, University College London, London, United Kingdom; m Department of Microbiology, Queen Mary Hospital, Hong Kong, People’s Republic of China

**Keywords:** SARS-CoV-2, variant of concern, Omicron variant, COVID-19 vaccine, neutralizing antibody

## Abstract

The novel SARS-CoV-2 Omicron variant may increase the risk of re-infection and vaccine breakthrough infections as it possesses key mutations in the spike protein that affect neutralizing antibody response. Most studies on neutralization susceptibility were conducted using specimens from adult COVID-19 patients or vaccine recipients. However, since the paediatric population has an antibody response to SARS-CoV-2 infection that is distinct from the adult population, it is critical to assess the neutralization susceptibility of pediatric serum specimens. This study compared the neutralization susceptibility of serum specimens collected from 49 individuals of <18 years old, including 34 adolescent BNT162b2 (Pfizer-BioNTech) vaccine recipients, and 15 recovered COVID-19 patients aged between 2 and 17. We demonstrated that only 38.2% of BNT162b2 vaccine recipients and 26.7% of recovered COVID-19 patients had their serum neutralization titre at or above the detection threshold in our live virus microneutralization assay. Furthermore, the neutralizing antibody titer against the Omicron variant was substantially lower than those against the ancestral virus or the Beta variant. Our results suggest that vaccine recipients and COVID-19 patients in the pediatric age group will likely be more susceptible to vaccine breakthrough infections or reinfections due to the Omicron variant than previous variants.

## Introduction

Although COVID-19 infections among children are generally milder than those seen in adults, severe complications, such as the multisystem inflammatory syndrome in children (MIS-C), and long-term sequelae, such as the “long COVID,” have been described [1–5]. Transmission from children to their adult household members has been shown to result in their hospitalization [6]. Furthermore, the COVID-19 pandemic also negatively impacted the psychosocial wellbeing of children and their families, particularly those with special education needs and lower socio-economical status [7].

COVID-19 vaccines are very effective, especially against severe diseases. These vaccines are largely safe in children [8,9], and are effective in reducing the transmission and complications against infection from the SARS-CoV-2 variants of concern (VOC), such as the Alpha, Beta, and Delta variants [10,11]. Recently, a new VOC, B.1.1.529, was identified in Botswana and South Africa in early November 2021 and was designated as the Omicron variant by the World Health Organization soon afterwards. The Omicron variant exhibits higher transmissibility when compared with the Delta variant [12]. Several studies, including our own study, have recently shown that serum specimens collected from vaccine recipients failed to neutralize the Omicron variant [13,14]. However, antibody response in children is distinct from those of adults [15]. Pediatric patients with prior COVID-19 infection had reduced neutralizing antibody response when compared with adults [15,16], whereas adolescents have a more robust neutralizing antibody response than adults in clinical trials of BNT162b2 and Coronavac [17,18]. Hence, it is critical to specifically assess whether the immune sera from children can neutralize the Omicron variant. This study aimed to determine the susceptibility of the Omicron variant to serum antibodies from children who recovered from COVID-19 infection or those who have received two doses of the BNT162b2 vaccines.

## Methods

### Patient recruitment

The COVID-19 vaccine recipient cohort consisted of archived serum specimens from BNT162b2 recipients that were collected from subjects who were suspected or confirmed to develop myocarditis, pericarditis, or other adverse events following two doses of the BNT162b2 vaccines and have been previously described [19]. Subjects who developed myocarditis or pericarditis after the first dose of the BNT162b2 vaccine were excluded.

The recovered COVID-19 patient cohort consisted of archived serum specimens from children who have recovered from laboratory-confirmed COVID-19 infections and were collected in our previous study [20]. Since our aim is to assess the fold reduction neutralization antibody titer against the Omicron variant when compared with that against the ancestral virus, only those with a neutralization antibody titer of ≥80 against the ancestral virus were selected.

This study was approved by the Institutional Review Board of the University of Hong Kong/Hospital Authority Hong Kong West Cluster (Reference: UW 20-292 and UW 21-548), the Kowloon West Cluster Research Ethics Committee [Reference: KW/FR-20-086(148-10)], and the Hospital Authority Central Institutional Review Board (CIRB-2021-003-4)

### Live virus microneutralization (MN) assay

Live virus MN assay was performed in a biosafety level 3 facility as we described previously [13,21]. Serum specimens were heat inactivated at 56°C for 30 min and were serially diluted in 2-folds starting from 1:10. Duplicates of each diluted serum were mixed with 100 TCID_50_ of an ancestral lineage A virus (GISAID accession number: EPI_ISL_434571), a Beta variant virus (GISAID accession number: EPI_ISL_2423556), and an Omicron variant virus (GISAID accession number EPI_ISL_7138045) for 1 hour, and the serum-virus mixture was then added to VeroE6/TMPRSS2 cells. Cytopathic effect was examined on day 3 after virus inoculation. The live virus microneutralization antibody titer was determined as the highest dilution with 50% inhibition of cytopathic effect.

### Statistical analysis

Statistical analysis was performed using SPSS 26.0 (IBM SPSS Statistics) and GraphPad PRISM 9.1.1 (GraphPad Software, San Diego CA, USA). Log-transformed microneutralization antibody titres against different variants were compared using one-way ANOVA with Tukey’s multiple comparisons test. Log-transformed fold reduction was compared using Wilcoxon matched-pairs signed-rank test. For the purpose of statistical analysis, an MN titer of <10 was considered as 5. A *P* value of <0.05 was considered statistically significant.

## Results

This study included serum specimens from a total of 49 individuals, of whom 34 were COVID-19 vaccine recipients, and 15 were recovered COVID-19 patients. All COVID-19 vaccine recipients were aged 12 years or above, because children aged 11 years or below were not included in the recommended group for the COVID-19 vaccine in Hong Kong SAR at the time of writing. All have received two doses of BNT162b2 vaccine, and the serum samples were collected at a median of 26.5 days after the first dose (range 24–87 days), and at a median of four days after the second dose (range 3–65 days). For recovered COVID-19 patients, their age ranged from 2.6 to 17.9 years old (Table 1). These recovered patients were first diagnosed to have COVID-19 between November 2020 and January 2021, and their serum samples were collected at a median of 44 days after the first laboratory diagnosis (range 29–96 days). Four of these patients were infected with the B.1.36.27 lineage. The virus lineage information is not available for the 11 other patients, but they are likely infected with the B.1.36.27 lineage, which was the main lineage circulating in Hong Kong during this period [22].

**Table 1.** Demographics and clinical information of recovered COVID-19 patients and vaccine recipients.

	Vaccine recipients (*n* = 34)	Recovered COVID-19 patients (*n* = 15)	*P* value
Female sex, no. (%)	6 (17.6%)	10 (66.7%)	0.002^a^
Median age in years (range)	15.0 (12.7–17.9)	9.6 (2.6–17.9)	0.001^b^
Symptomatic, no. (%)	N/A	14 (93.3%)	N/A

Note: N/A, Not applicable.

^a^ Fisher’s exact test.

^b^ Mann Whitney U test.

All recovered COVID-19 patients and vaccine recipients had an MN titer of ≥10 against the ancestral lineage A virus. Similarly, 94.1% (32/34) of vaccine recipients and all recovered patients had MN titer of ≥10 against the Beta variant. However, only 38.2% (13/34) and 26.7% (4/15) of vaccine recipients and recovered patients had an MN titer of ≥10 against the Omicron variant, respectively.

**Figure 1.**
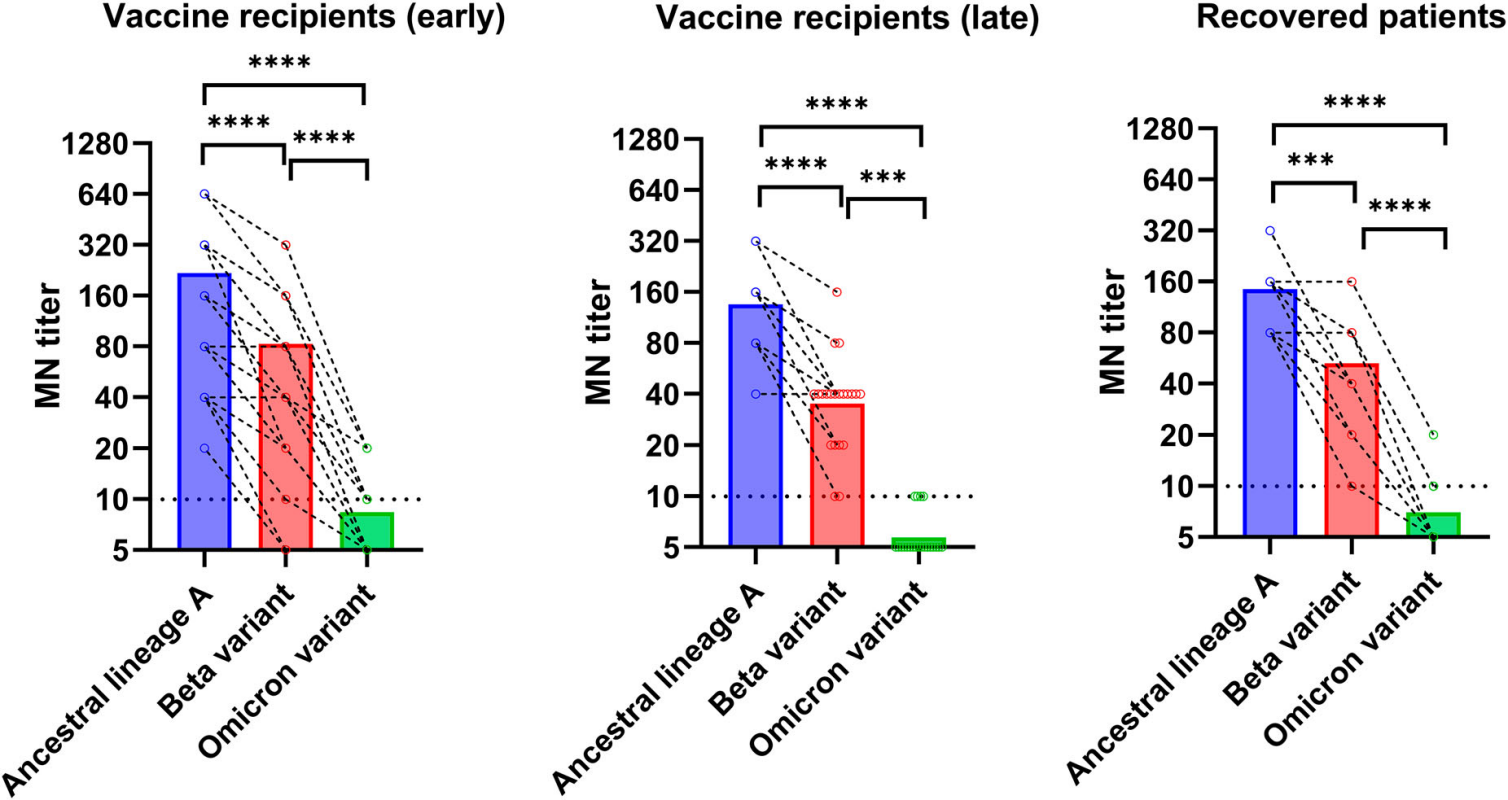
Comparison of microneutralization antibody (MN) titers between the Omicron variant and other variants or ancestral SARS-CoV-2 virus. Vaccine recipients (early): serum specimens collected from 34 vaccine recipients at a median of four days after the 2nd dose. Vaccine recipients (late): serum specimens collected from 21 vaccine recipients at a median of 44 days after the 2nd dose. Open circles represent the MN titer of each serum specimen. The MN titers from the same patient were connected by the dotted line. *** *P* < 0.001; **** *P* < 0.0001.

**Figure 2.**
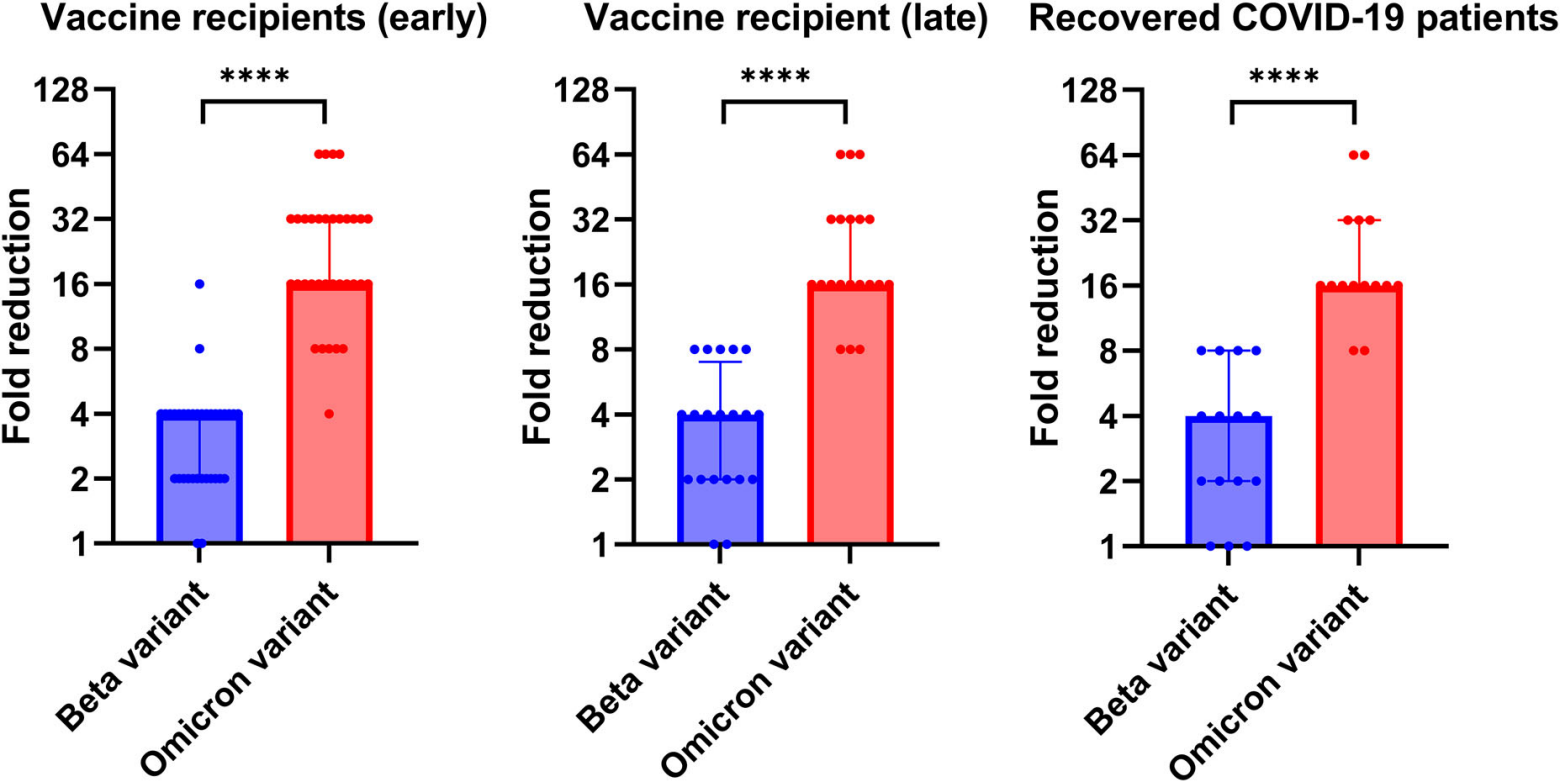
Fold reduction of microneutralization antibody titers when compared with ancestral lineage A virus. Median and interquartile range are shown.

The geometric mean microneutralization antibody titer (GMT) against the Omicron variant (Vaccine recipients: 7.2 [95% CI, 6.0–8.6]; Recovered patients: 6.3 [95% CI, 5.0–8.0]) was significantly lower than those against the ancestral virus (Vaccine recipients: 150.5 [95% CI, 109.6–206.7]; Recovered patients:127.0 [95% CI, 96.2–167.7]) or the Beta variant (Vaccine recipients: 49.05 [95% CI, 33.6–71.7]; Recovered patients: 41.9 [95% CI, 28.2–62.3]) for both vaccine recipients and recovered COVID-19 patients (Figure 1). Relative to the ancestral lineage A virus, there was a significantly greater fold reduction for Omicron variant than those for the Beta variant for both vaccine recipients and recovered patients (*P* < 0.0001) (Figure 2). However, there was no statistically significant difference in the fold reduction between vaccine recipients and recovered patients for both Beta and Omicron variants.

For the vaccine recipient cohort, we also assessed whether the differences between the ancestral virus and the variants were maintained in the long-term. Late serum specimens were available for 21 vaccine recipients, which were collected at a median of 65 days after the first dose (range 36–146 days) and at a median of 44 days after the second dose (range 15–124 days). While there was a decline in antibody titer against the ancestral lineage A virus, Beta variant, and Omicron variant in the late serum specimens (Figure 1), the fold reduction of Beta variant and Omicron variant relative to ancestral lineage A virus was similar to those of the early serum specimens (Figure 2).

## Discussions

To date, this is the first study demonstrating a reduced susceptibility of Omicron variant to serum antibodies from children who recovered from COVID-19 infection or those who have received two doses of the BNT162b2 vaccines. Since the Omicron variant carries several spike mutations that are known to confer resistance to neutralizing antibodies, many studies have been conducted rapidly to assess the degree of resistance associated with this novel variant. However, these studies were conducted either in adults, or the age group was not specified [13,14,23–25]. Our previous study showed that adult BNT162b2 vaccine recipients had a 36-40-fold reduction in MN titer against the Omicron variant [13]. However, results from the current adolescent post-vaccination cohort showed only a 20.9 fold reduction in GMT, or a median of 16-fold reduction. This can be related to the difference in the quality of antibody response between adolescents and adults. A previous study found that antibody in the pediatric age group are mainly targeting the S protein, while antibody from adults target both S and N protein [15].

Our previous paediatric study showed that the antibody levels in children recovered from COVID-19 infection had a half-life of 121.6 days and an estimated duration of 7.9 months [20]. Hence, the antibody levels of majority of the paediatric COVID-19 patients would have dropped to a very low level, if not undetectable, beyond 8 months after infection. Further studies are required to determine whether vaccination after prior COVID-19 infection in children would provide added protection against re-infection and breakthrough infection by the Omicron variant.

This study has several limitations. First, as many patients had a MN titer of <10, their fold reduction may have been underestimated. Second, this study did not assess cross-reactive T cell immunity against the Omicron variant. Third, since COVID-19 vaccine was not yet recommended for children aged 11 or younger in Hong Kong at the time of writing this manuscript, we were not able to assess the effect of Omicron variant in this age group. Fourth, this study only included BNT162b2 vaccine recipients. Data is required for other vaccine types. Finally, vaccine recipients in this cohort may have a dysregulated humoral immune response. However, none of the patients received immunosuppressants, such as steroids, intravenous immunoglobulins, or biologics [19].

Neutralization antibody titer has been shown to correlate with vaccine effectiveness and protection from re-infection [26,27]. Our results suggest that the effectiveness of COVID-19 vaccines may be reduced for the Omicron variant in the paediatric age group. In order to achieve a better protection against the Omicron variant, adolescents who have received two doses of COVID-19 vaccines may require a third dose, and recovered pediatric COVID-19 patients should receive COVID-19 vaccines. However, since mRNA vaccines are associated with an increased risk of myocarditis in adolescents [19,28], there must be a careful balance between adverse events and vaccine effectiveness when choosing the type of vaccine for the third dose for this age group.

## Supplementary Material

Supplemental Material

## References

[1] Chua GT, Xiong X, Choi EH, et al. COVID-19 in children across three Asian cosmopolitan regions. Emerg Microbes Infect. 2020;9:2588–2596. 33138739 10.1080/22221751.2020.1846462 PMC7723019

[2] Chua GT, Wong JSC, Lam I, et al. Clinical characteristics and transmission of COVID-19 in children and youths during 3 waves of outbreaks in Hong Kong. JAMA Netw Open. 2021;4:e218824. 33938934 10.1001/jamanetworkopen.2021.8824 PMC8094012

[3] Xiong X, Chua GT, Chi S, et al. A comparison between Chinese children infected with coronavirus disease-2019 and with severe acute respiratory syndrome 2003. J Pediatr. 2020;224:30–36. 32565097 10.1016/j.jpeds.2020.06.041 PMC7301144

[4] Feldstein LR, Tenforde MW, Friedman KG, et al. Characteristics and outcomes of US children and adolescents With multisystem inflammatory syndrome in children (MIS-C) compared with severe acute COVID-19. JAMA. 2021;325:1074–1087. 33625505 10.1001/jama.2021.2091 PMC7905703

[5] Hageman JR. Long COVID-19 or post-acute sequelae of SARS-CoV-2 infection in children, adolescents, and young adults. Pediatr Ann. 2021;50:e232–e233. 34115558 10.3928/19382359-20210519-02

[6] Chu VT, Yousaf AR, Chang K, et al. Household transmission of SARS-CoV-2 from children and adolescents. N Engl J Med. 2021;385:954–956. 34289272 10.1056/NEJMc2031915 PMC8314736

[7] Tso WWY, Wong RS, Tung KTS, et al. Vulnerability and resilience in children during the COVID-19 pandemic. Eur Child Adolesc Psychiatry. 2020;1–16. DOI:10.1007/s00787-020-01680-8. 33205284 PMC7671186

[8] Walter EB, Talaat KR, Sabharwal C, et al. Evaluation of the BNT162b2 Covid-19 vaccine in children 5 to 11 years of age. N Engl J Med. 2022;386:35–46. 34752019 10.1056/NEJMoa2116298 PMC8609605

[9] Wu Z, Hu Y, Xu M, et al. Safety, tolerability, and immunogenicity of an inactivated SARS-CoV-2 vaccine (CoronaVac) in healthy adults aged 60 years and older: a randomised, double-blind, placebo-controlled, phase 1/2 clinical trial. Lancet Infect Dis. 2021;21:803–812. 33548194 10.1016/S1473-3099(20)30987-7 PMC7906628

[10] Reis BY, Barda N, Leshchinsky M, et al. Effectiveness of BNT162b2 vaccine against delta variant in adolescents. N Engl J Med. 2021;385:2101–2103. 34670036 10.1056/NEJMc2114290 PMC8552532

[11] Li XN, Huang Y, Wang W, et al. Effectiveness of inactivated SARS-CoV-2 vaccines against the delta variant infection in Guangzhou: a test-negative case-control real-world study. Emerg Microbes Infect. 2021;10:1751–1759. 34396940 10.1080/22221751.2021.1969291 PMC8425710

[12] Agency UHS. SARS-CoV-2 variants of concern and variants under investigation in England. Technical Briefing 32. Available from: https://assets.publishing.service.gov.uk/government/uploads/system/uploads/attachment_data/file/1042046/Technical_Briefing_32.pdf

[13] Lu L, Mok BW, Chen LL, et al. Neutralization of SARS-CoV-2 Omicron variant by sera from BNT162b2 or Coronavac vaccine recipients. Clin Infect Dis. 2021;ciab1041. DOI:10.1093/cid/ciab1041. 34915551 PMC8754807

[14] Zhang L, Li Q, Liang Z, et al. The significant immune escape of pseudotyped SARS-CoV-2 variant Omicron. Emerg Microbes Infect. 2022;11:1–5. 34890524 10.1080/22221751.2021.2017757 PMC8725892

[15] Weisberg SP, Connors TJ, Zhu Y, et al. Distinct antibody responses to SARS-CoV-2 in children and adults across the COVID-19 clinical spectrum. Nat Immunol. 2021;22:25–31. 33154590 10.1038/s41590-020-00826-9 PMC8136619

[16] Pierce CA, Preston-Hurlburt P, Dai Y, et al. Immune responses to SARS-CoV-2 infection in hospitalized pediatric and adult patients. Sci Transl Med. 2020;12:eabd5487. 32958614 10.1126/scitranslmed.abd5487 PMC7658796

[17] Frenck RW J, Klein NP, Kitchin N, et al. Safety, immunogenicity, and efficacy of the BNT162b2 Covid-19 vaccine in adolescents. N Engl J Med. 2021;385:239–250. 34043894 10.1056/NEJMoa2107456 PMC8174030

[18] Han B, Song Y, Li C, Yang W, Ma Q, Jiang Z, et al. Safety, tolerability, and immunogenicity of an inactivated SARS-CoV-2 vaccine (CoronaVac) in healthy children and adolescents: a double-blind, randomised, controlled, phase 1/2 clinical trial. Lancet Infect Dis 2021;21:1645–1653. 34197764 10.1016/S1473-3099(21)00319-4 PMC8238449

[19] Chua GT, Kwan MYW, Chui CSL, et al. Epidemiology of acute myocarditis/pericarditis in Hong Kong adolescents following comirnaty vaccination. Clin Infect Dis. 2021;ciab989. DOI:10.1093/cid/ciab989. 34849657 PMC8767823

[20] Tsang HW, Chua GT, To KK, et al. Assessment of SARS-CoV-2 immunity in Convalescent children and adolescents. Front Immunol. 2021;12:797919. 34975908 10.3389/fimmu.2021.797919 PMC8718543

[21] Lu L, Chu AW, Zhang RR, et al. The impact of spike N501Y mutation on neutralizing activity and RBD binding of SARS-CoV-2 convalescent serum. EBioMedicine. 2021;71:103544. 34419925 10.1016/j.ebiom.2021.103544 PMC8374549

[22] Chan WM, Ip JD, Chu AW, et al. Phylogenomic analysis of COVID-19 summer and winter outbreaks in Hong Kong: An observational study. Lancet Reg Health West Pac. 2021;10:100130. 33778795 10.1016/j.lanwpc.2021.100130 PMC7985010

[23] Dejnirattsiai W, Shaw RH, Supasa P, et al. Reduced neutralisation of SARS-CoV-2 omicron B.1.1.529 variant by post-immunisation serum. Lancet. 2022;399:234–236. 34942101 10.1016/S0140-6736(21)02844-0 PMC8687667

[24] Cameroni E, Bowen JE, Rosen LE, et al. Broadly neutralizing antibodies overcome SARS-CoV-2 Omicron antigenic shift. Nature. 2021. DOI:10.1038/s41586-021-04386-2. PMC9531318 35016195

[25] Rossler A, Riepler L, Bante D, et al. SARS-CoV-2 Omicron variant neutralization in serum from vaccinated and Convalescent persons. N Engl J Med. 2022:NEJMc2119236. DOI:10.1056/NEJMc2119236. PMC8781314 35021005

[26] Gilbert PB, Montefiori DC, McDermott AB, et al. Immune correlates analysis of the mRNA-1273 COVID-19 vaccine efficacy clinical trial. Science. 2022;375:43–50. 34812653 10.1126/science.abm3425 PMC9017870

[27] Lumley SF, O'Donnell D, Stoesser NE, et al. Antibody status and incidence of SARS-CoV-2 infection in health care workers. N Engl J Med. 2021;384:533–540. 33369366 10.1056/NEJMoa2034545 PMC7781098

[28] Mevorach D, Anis E, Cedar N, et al. Myocarditis after BNT162b2 mRNA vaccine against Covid-19 in Israel. N Engl J Med. 2021;385:2140–2149. 34614328 10.1056/NEJMoa2109730 PMC8531987

