# Supplementary figures and images for: Omicron variant susceptibility to neutralizing antibodies induced in children by natural SARS-CoV-2 infection or COVID-19 vaccine
Source: Emerg Microbes Infect. 2022 Feb 10;11(1):543–7. doi: 10.1080/22221751.2022.2035195 (PMC8843159; doi:10.1080/22221751.2022.2035195)

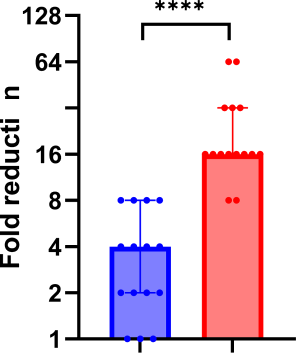
Vaccine recipients (early) Vaccine recipient (late) Recovered COVID-19 patients


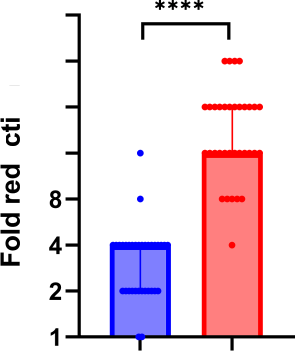

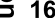


128

o 32


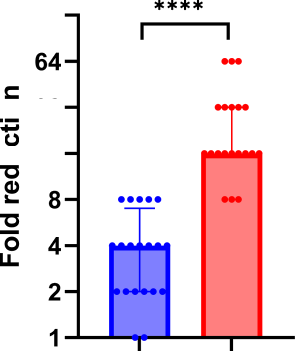

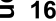


128

o 32

#
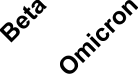

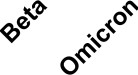
q,°

O’

Supplement: Supplemental Material [file TEMI_A_2035195_SM3838.docx]
